# Sensitization of knee-innervating sensory neurons by tumor necrosis factor-α-activated fibroblast-like synoviocytes: an in vitro, coculture model of inflammatory pain

**DOI:** 10.1097/j.pain.0000000000001890

**Published:** 2020-04-22

**Authors:** Sampurna Chakrabarti, Zoe Hore, Luke A. Pattison, Sylvine Lalnunhlimi, Charity N. Bhebhe, Gerard Callejo, David C. Bulmer, Leonie S. Taams, Franziska Denk, Ewan St. John Smith

**Affiliations:** a Department of Pharmacology, University of Cambridge, Cambridge, United Kingdom; b Wolfson Centre for Age-Related Diseases, Institute of Psychiatry, Psychology and Neuroscience, King's College London, London, United Kingdom,; c Department of Inflammation Biology, Centre for Inflammation Biology and Cancer Immunology, School of Immunology and Microbial Sciences, King's College London, London, United Kingdom

**Keywords:** Synoviocytes, Sensory neurons, Pain, Inflammation, Tumor necrosis factor, Complete Freund's adjuvant, Knee

## Abstract

Pain is a principal contributor to the global burden of arthritis with peripheral sensitization being a major cause of arthritis-related pain. Within the knee joint, distal endings of dorsal root ganglion neurons (knee neurons) interact with fibroblast-like synoviocytes (FLS) and the inflammatory mediators they secrete, which are thought to promote peripheral sensitization. Correspondingly, RNA sequencing has demonstrated detectable levels of proinflammatory genes in FLS derived from arthritis patients. This study confirms that stimulation with tumor necrosis factor (TNF-α) results in expression of proinflammatory genes in mouse and human FLS (derived from osteoarthritis and rheumatoid arthritis patients), as well as increased secretion of cytokines from mouse TNF-α-stimulated FLS (TNF-FLS). Electrophysiological recordings from retrograde labelled knee neurons cocultured with TNF-FLS, or supernatant derived from TNF-FLS, revealed a depolarized resting membrane potential, increased spontaneous action potential firing, and enhanced TRPV1 function, all consistent with a role for FLS in mediating the sensitization of pain-sensing nerves in arthritis. Therefore, data from this study demonstrate the ability of FLS activated by TNF-α to promote neuronal sensitization, results that highlight the importance of both nonneuronal and neuronal cells to the development of pain in arthritis.

## Abstract 2

Supplemental Digital Content is Available in the Text.

Fibroblast-like synoviocytes (FLS) interact with articular sensory nerve endings. Coculture of tumor necrosis factor (TNF-α)-stimulated FLS and knee-innervating sensory neurons reveals FLS-mediated neuronal sensitization.

## 1. Introduction

Joint inflammation and pain are the major clinical symptoms of arthritis. Inflammation is part of the body's immune response to tissue damage and involves multiple cell types, including leukocytes and synoviocytes in synovial joints. These nonneuronal cells are either in direct contact with, or in close proximity to, the distal endings of joint-innervating dorsal root ganglion (DRG) sensory neurons, and this interaction is thought to cause peripheral sensitization and development of inflammatory pain.

During inflammatory diseases such as rheumatoid arthritis (RA), the affected joints undergo hyperplasia due to leukocyte infiltration and fibroblast-like synoviocyte (FLS) proliferation. Fibroblast-like synoviocytes are key effectors in RA as they become active upon stimulation by inflammatory cytokines (released by macrophage-like synoviocytes and T-lymphocytes) and secrete matrix metalloproteases (MMP) that cause joint destruction.^9^ Correspondingly, single-cell transcriptional analysis of human RA-FLS has identified distinct “destructive” and “inflammatory” FLS subgroups.^17^ In addition, FLS can also support and maintain the ongoing inflammation in arthritic joints by secreting proinflammatory mediators themselves.^9^ To better understand the inflammatory phenotype of FLS, several studies have used cytokine stimulation (eg, interleukin 1β, IL-1β, or tumor necrosis factor-α, TNF-α) to robustly induce inflammation.^14,27,30,40^ “Inflamed” FLS have been used in coculture systems to dissect the complex interaction between FLS and other cell types in the joint environment. Coculture of human RA-FLS with T-lymphocytes^7,11,37,67^ or macrophages/monocytes^6,8,15^ has been found to increase the concentration of prostaglandin E_2_, IL-6, IL-8, MMP-1, and MMP-3 in the culture medium. Furthermore, upregulation of these proinflammatory mediators in culture supernatants was inhibited by anti-TNF-α^11,55^ and anti-IL-6 antibodies.^55^

However, most coculture studies have focused on immune interactions during joint inflammation and therefore, the communication between neurons and synoviocytes is much less understood. Fibroblast-like synoviocytes have been hypothesized to enhance nociceptive responses^64^ and alter the biomechanical microenvironment of DRG neurons.^31^ Fibroblast-like synoviocytes derived from chronic antigen-induced arthritic (AIA) rats were observed to increase the expression of receptors associated with nociception, namely neurokinin 1, bradykinin 2, and transient receptor potential vanilloid 1 (TRPV1) in DRG neurons when cocultured.^4^ However, this study did not functionally assess modulation of DRG neuron excitability, which is a key mechanism of peripheral sensitization and hence pain. Indeed, we recently showed that human osteoarthritic (OA) synovial fluid (a lubricating fluid largely secreted by FLS^5^) causes hyperexcitability of murine sensory neurons and increased TRPV1 function.^12^

To establish a suitable proinflammatory phenotype for our cultures, we used primary FLS derived from mice undergoing acute, unilateral complete Freund's adjuvant (CFA)-induced knee inflammation, which can produce neuronal hyperexcitability.^13^ We also tested primary mouse knee-derived FLS treated with TNF-α, one of the main cytokines upregulated in CFA-injected mouse tissues^65^ and in inflammatory arthritis,^53^ and validated our results in FLS derived from human OA and RA patients. The proinflammatory phenotype of FLS was established to test the hypothesis that after induction of inflammation, mouse knee-derived FLS will increase excitability and TRP function of knee-innervating DRG neurons (knee neurons) in an FLS-DRG neuron coculture system.

## 2. Methods

### 2.1. Animals

All mice used in this study were 6- to 12-week-old C57BL/6J mice (Envigo, Indianapolis, IN) randomly allocated to each experiment. Unless otherwise stated, female mice were used because in humans, females are at a higher risk for arthritic pain.^28,51,62^ Mice were housed in groups of up to 5 in a temperature controlled (21°C) room with appropriate bedding materials, a red shelter, and enrichment. They were on a 12-hour light/dark cycle with food and water available ad libitum. Experiments in this study were regulated under the Animals (Scientific Procedure) Act 1986, Amendment Regulations 2012. All protocols were approved by a U.K. Home Office project license granted to Dr Ewan St. John Smith (P7EBFC1B1) and reviewed by the University of Cambridge Animal Welfare and Ethical Review Body.

### 2.2. Knee injections

Under anesthesia (100 mg/kg ketamine and 10 mg/kg xylazine, intraperitoneally), mice were injected intra-articularly through the patellar tendon into each knee with the retrograde tracer, fast blue (FB, 1.5 μL 2% in 0.9% saline, Polysciences) or into the left knee with 7.5 μL CFA (10 mg/mL, Chondrex). Vernier calipers were used to measure knee width (as before^13^), both before and 24 hours after CFA injection.

### 2.3. Isolation and culture of fibroblast-like synoviocytes

Twenty-four hours after CFA injection into the knee, mice were killed by cervical dislocation and decapitation. Knee joints were exposed by removing the skin, and the quadriceps muscles were resected in the middle and pulled distally to expose the patellae. Patellae were then collected by cutting through the surrounding ligaments, as described before,^25^ in phosphate-buffered saline (PBS) and then transferred into one well of a 24-well plate with FLS culture media containing: Dulbecco's Modified Eagle Medium F-12 Nutrient Mixture (Ham) (Life Technologies), 25% fetal bovine serum (Sigma), 2 mM glutamine (Sigma), and 100 mg/mL penicillin/streptomycin (Life Technologies). Cells took approximately 10 days to grow to 70% confluency; medium was changed every other day. For P1, FLS were trypsinized with 1% trypsin (Sigma), resuspended in FLS culture media and transferred into 2 wells of a 6-well plate. Fibroblast-like synoviocytes from 2 animals were combined at P2. For subsequent passages, FLS were transferred into 60-mm dishes. Contralateral (Contra) and CFA-injected knees/cells (Ipsi) were kept separate at all stages. The cells were maintained in a humidified, 37°C, 5% CO_2_ incubator. Fibroblast-like synoviocytes were also cultured from mice that had not undergone any knee CFA injection (control) until P5. A random selection of these dishes, from 3 separate biological replicates, was incubated for 24 to 48 hours (as per experimental design) in culture medium with recombinant mouse TNF-α (10 ng/mL from a stock solution of 100 μg/mL made up in 0.2% bovine serum albumin and sterile PBS, R&D systems, aa-80325) to stimulate release of inflammatory mediators (TNF-FLS).^29^

### 2.4. Culture of raw 264.7 cells

Raw 264.7 cells (EACC) were cultured in medium containing Dulbecco's Modified Eagle Medium F-12 Nutrient Mixture (Ham) (Life Technologies), 10% fetal bovine serum (Sigma), 2 mM glutamine (Sigma), and 100 mg/mL penicillin/streptomycin (Life Technologies). Cells were maintained in a humidified 37°C, 5% CO_2_ incubator. This macrophage-like cell line^59^ was used to assess the level of macrophage contamination in FLS cultures.

### 2.5. RNA extraction and reverse transcriptase–quantitative/polymerase chain reaction (RT-q/PCR) of mouse fibroblast-like synoviocytes

For all conditions, RNA was extracted from two 60-mm dishes (Thermo Fisher) of FLS (various passages) and from one T-25 flask (Greiner Bio-one, Kremsmünster, Austria) of Raw 264.7 cells at P3 using the RNeasy Mini Kit (Qiagen). 500 ng of the extracted RNA was used to synthesize cDNA using a High-Capacity cDNA RT kit (Applied Biosystems) following the manufacturer's guidelines, using a T100 Thermal Cycler (Bio-Rad). The resultant cDNA was diluted to a 1:5 ratio with nuclease-free water, and quantitative PCR (qPCR) was performed using a StepOnePlus Real-Time PCR system, following the manufacturer's guidelines on settings (Applied Biosystems) using TaqMan probes (Thermo Fisher) (Supplementary Table 1 listing genes of interest analyzed in this study, available at http://links.lww.com/PAIN/A1000). The fluorescence intensity of samples was captured during the last minute of each cycle. All reactions were run in triplicate with appropriate negative controls with water containing no cDNA.

For RT-qPCR reactions, data were obtained as Ct values (the cycle number at which fluorescent signals emitted by the TaqMan probe crossed a threshold value). Only Ct values below 35 were analyzed to determine ΔCt values by subtracting the Ct of *18S* ribosomal RNA from the Ct of target gene.^41^ ΔCt values of target genes were subtracted from average ΔCt values of their controls to calculate ΔΔCt, followed by 2^ (−ΔΔCt) to calculate fold change.^39^

Fibroblast-like synoviocyte gene expression was also assessed by RT-PCR. DreamTaq Polymerase (Thermo Fisher) was used to amplify a section within the open reading frames of various genes from 5 ng template cDNA. The sequences of designed oligonucleotides (Sigma) are listed in Supplementary Table 1 (available at http://links.lww.com/PAIN/A1000). Negative controls (using water/RNA as the template) were performed for each biological sample with a randomly selected primer pairing. PCR products were resolved on 2% agarose containing 1X GelRed Nucleic Acid Stain (Biotum) and imaged with a GeneFlash Gel Documentation System (Syngene). A randomly selected subset of reactions was repeated with 2.5 ng template cDNA to ensure reproducibility. Densitometry analyses were performed using ImageJ Software (NIH) where relative expression was determined by dividing the band intensity of each gene by that of the housekeeping gene, *18S* ribosomal RNA, for each biological replicate.

### 2.6. Cytokine antibody array

Before RNA extraction, 2 mL of culture media from P5 Contra, Ipsi, control, and TNF-FLS (48-hour) were collected and stored at −80°C until use. Culture medium was pooled from 3 cultures for each of the 4 conditions and assayed (undiluted) for the presence of 40 inflammatory mediators using Mouse Inflammatory Antibody Array Membranes (ab133999; Abcam) according to the manufacturer's instructions. Chemiluminescence was imaged using a BioSpectrum 810 imaging system (UVP) with 3-minute exposure. Location of the 40 cytokines detected by the array membranes are shown in Supplementary Table 2 (available at http://links.lww.com/PAIN/A1000).

Densitometry of the spots in the array membranes was performed using ImageJ software (NIH). Briefly, the mean gray value of each spot was measured from all membranes using the same circular region of interest. The spots of interests were then subtracted from background (average of all negative control spots) and normalized to the positive control spots of the reference membrane (control FLS media). A fold change value was obtained by dividing normalized intensities of the membrane of interest and the control membrane, analyte-by-analyte.

### 2.7. Culture of human fibroblast-like synoviocytes

Fibroblast-like synoviocytes were obtained from 1 OA and 3 RA female patients (age range 61-81 years, see Supplementary Table 3 for detailed patient information, available at http://links.lww.com/PAIN/A1000) with approval from local ethics committee (07/H0809/35). These were then seeded separately at 10,000 cells/well in 200 µL of DMEM media (Sigma) supplemented with 10% FCS (Fisher Scientific), 100 units/mL of penicillin/streptomycin (Fisher Scientific), 2% glutamine (Thermo Fisher), and Fungizone (Thermo Fisher) into a 96-well flat bottom plate (Starlab). The plate was incubated overnight at 37°C to allow for SF attachment and the following morning, media was replaced with either 200 µL of fresh DMEM containing 10 ng/mL of TNF-α (PeproTech), or fresh DMEM alone. Supernatants were collected after 24 hours of incubation at 37°C.

### 2.8. RNA extraction and RT-quantitative PCR of human fibroblast-like synoviocytes

RNA was extracted using a RNeasy Micro Kit (Qiagen) according to the manufacturer's protocol, and then reverse transcribed and amplified using a slightly modified version of the Smart-seq2 method.^48^ Briefly, in a RNase/DNA-free fume hood, 2 µL of RNA was mixed with 1 µL of dNTP mix (Thermo Scientific), 1 µL of Oligo-dT primer (Merck), and 5.7 µL of RT mix, and then reverse transcribed on a PTC-225 Gradient Thermal Cycler (MJ Research). 15 µL of PCR mix was added to the resulting cDNA and amplified according to previously established protocol. Concentrations were checked on a Qubit 3.0 (Invitrogen), with values ranging from 20.4 to 45.0 ng/µL. Samples were diluted to 1 ng/µL with double-distilled H_2_O (ddH_2_O), and 1 ng was used for standard SYBR Green (Roche) RT-qPCR reactions on a LightCycler480 (Roche) to probe for the genes of interest (see Supplementary Table 1 for primer sequences, available at http://links.lww.com/PAIN/A1000). All primers were tested for their efficiency and specificity. The mean of housekeeping genes *B2M* and *Ywhaz* was used to calculate ΔCt values. All reactions were run in duplicate with water used as a negative control.

### 2.9. Dorsal root ganglion neuron isolation and culture

Lumbar DRG (L2-L5, those that primarily innervate the knee) were collected from FB labelled mice 7 to 10 days after knee injections in ice-cold dissociation media containing L-15 Medium (1X) + GlutaMAX-l (Life Technologies), supplemented with 24 mM NaHCO_3_. DRG were then enzymatically digested in type 1A collagenase (Sigma) and trypsin solution (Sigma) at 37°C, followed by mechanical trituration as described before.^13^ Dissociated DRG neurons were plated onto poly-D-lysine and laminin-coated glass-bottomed dishes (MatTek, P35GC-1.5-14-C) and cultured either on their own (monoculture), on a layer of FLS (coculture, see below) or in 48-hour conditioned media from TNF-FLS for 24 hours. The DRG culture medium contained L-15 Medium (1X) + GlutaMAX-l, 10% (v/v) fetal bovine serum, 24 mM NaHCO_3_, 38 mM glucose, and 2% (v/v) penicillin/streptomycin.

### 2.10. Dorsal root ganglion neuron/fibroblast-like synoviocyte coculture

For coculture studies, FLS were plated onto MatTek dishes and cultured for 24 hours with FLS culture media (with or without TNF-α stimulation). The next day, medium was removed from FLS plates, and DRG neurons were isolated as described above and plated on top of the FLS. Coculture plates were then kept in DRG culture medium for up to 24 hours for electrophysiological recording.

### 2.11. Cell staining

#### 2.11.1. Fibroblast-like synoviocyte immunocytochemistry

Fibroblast-like synoviocytes were plated overnight in wells of a 24-well plate, fixed with Zamboni's fixative^57^ for 10 minutes, permeabilized with 0.05% Triton X-100, and blocked with antibody diluent (0.2% [vol/vol] Triton X-100, 5% [vol/vol] donkey serum, and 1% [vol/vol] bovine serum albumin in PBS) for 30 minutes. The cells were then incubated overnight at 4°C in 1:100 (in antibody diluent) anti-cadherin-11 antibody (CDH-11, rabbit polyclonal, Thermo Fisher, 71-7600). Cells were washed 3 times with PBS-Tween and incubated in the conjugated secondary antibody, anti-rabbit Alexa-568 (1:1000 in PBS, Thermo Fisher, A10042), for 1 hour at room temperature (21°C). The secondary antibody was washed off 3 times with PBS-Tween and the cells were incubated in the nuclear dye DAPI (1:1000 in PBS, Sigma, D9452) for 10 minutes. Cells were further washed with PBS-Tween once and imaged in PBS using an EVOS FLoid Cell Imaging Station (Thermo Fisher) at 598 nm (for CDH-11) and 350 nm (for DAPI) wavelengths of light. Cells without primary antibody did not show fluorescence.

#### 2.11.2. Dorsal root ganglion neuron/fibroblast-like synoviocyte coculture live cell stain

To visualize DRG neuron/FLS coculture, live cell imaging was performed. Fibroblast-like synoviocytes were plated on MatTek dishes with 1:1000 (diluted in FLS culture medium) CellTracker Deep Red Dye (Thermo Fisher, C34565) and incubated for 24 hours in a humidified 37°C, 5% CO_2_ incubator. Dissociated DRG neurons (see above) were incubated in CellTracker Green Dye (1: 1000 diluted in DRG culture media, Thermo Fisher, C7025) for 15 minutes at room temperature (21°C), centrifuged (16000 g, 3 minutes, 5415R, Eppendorf), and resuspended in fresh medium. The FLS dishes were then washed twice with PBS, and the neuronal suspension was plated on top of the FLS monolayer and incubated overnight in the incubator. The coculture dishes were washed with PBS and imaged the following day using an Olympus BX51 microscope and QImaging camera at 650 nm (for deep red dye) and 488 nm (for green dye) wavelengths of light.

### 2.12. Whole-cell patch-clamp electrophysiology

Dorsal root ganglion neurons were bathed in extracellular solution containing (ECS, in mM): NaCl (140), KCl (4), MgCl_2_ (1), CaCl_2_ (2), glucose (4), and HEPES (10) adjusted to pH 7.4 with NaOH. Only FB-labelled neurons identified by their fluorescence upon excitation with a 365-nm LED (Cairn Research) were recorded. Patch pipettes of 5 to 10 MΩ were pulled with a P-97 Flaming/Brown puller (Sutter Instruments) from borosilicate glass capillaries and the intracellular solution used contained (in mM): KCl (110), NaCl (10), MgCl_2_ (1), EGTA (1), HEPES (10), Na_2_ATP (2), and Na_2_GTP (0.5) adjusted to pH 7.3 with KOH.

Action potentials (AP) were recorded in current clamp mode without current injection for 20 seconds (to investigate spontaneous AP firing) or after step-wise injection of 80-ms current pulses from 0 to 1050 pA in 50-pA steps using a HEKA EPC-10 amplifier (Lambrecht) and the corresponding Patchmaster software. Action potentials properties were analyzed using Fitmaster software (HEKA) or IgorPro software (Wavemetrics) as described before.^13^ Neurons were excluded from analysis if they did not fire an AP in response to current injection. For recording whole-cell voltage-clamp currents in response to TRP agonists capsaicin (1 μM from a 1 mM stock in ethanol, Sigma-Aldrich), cinnamaldehyde (100 μM from a 1 M stock in ethanol, Alfa Aesar), and menthol (100 μM from a 1 M stock in ethanol, Alfa Aesar), a 5-second baseline was established with ECS, followed by a 5-second randomized drug application. Peak drug response was measured in Fitmaster by subtracting the average of 2-second baseline immediately preceding the drug application and the maximum peak response reached during the 5 seconds of drug application. Peak current density is represented in graphs by dividing this peak response by the capacitance of the neuron. Data from at least 4 mice were used in all conditions (with each mouse being used for at least 2 conditions) and at least 3 neurons were recorded from each mouse in each category.

### 2.13. Ca^2+^ imaging

Fibroblast-like synoviocytes or DRG neurons were incubated with the Ca^2+^ indicator, Fluo-4 AM (10 μM diluted in ECS from a 10 mM stock solution in DMSO, Invitrogen), for 30 minutes at room temperature (21°C). Culture dishes were then washed and imaged using an inverted Nikon Eclipse T*i* microscope. Fluo-4 fluorescence was excited using a 470-nm LED (Cairn Research) and captured with a camera (Zyla cSMOS, Andor) at 1 Hz with an exposure time of 50 ms (for neurons) and 250 ms (for FLS) using Micro-Manager software (v1.4; NIH). Solutions were perfused in this system through a gravity-driven 12-barrel perfusion system.^20^ During imaging of neurons, MIP-1γ (10 and 100 ng/mL diluted in ECS from a stock concentration of 0.1 mg/mL in 0.1% bovine serum albumin/sterile PBS, Sigma) was applied for 20 seconds after establishing a baseline with ECS for 10 seconds. Neurons were allowed to recover for 5 minutes between drug applications. 50 mM KCl was used as a positive control.

During imaging of FLS, a 10-second baseline was established with ECS and then pH 4 to 7, and TRP agonists, 1 μM capsaicin, 100 μM cinnamaldehyde, and 100 μM menthol were applied for 10 seconds before a washout period of 4 minutes between drug applications. TRP agonist sensitivity and acid sensitivity was assessed on separate dishes. Three biological repeats were conducted on separate days for each condition. 10 μM ionomycin was used as a positive control after all FLS Ca^2+^ imaging experiments.

Analysis was conducted as described before.^12^ Briefly, mean gray values were extracted by manually drawing around FLS or neurons in the ImageJ software. These values were then fed into a custom-made R-toolbox (https://github.com/amapruns/Calcium-Imaging-Analysis-with-R.git) to compute the proportion of cells responding to each drug and their corresponding magnitude of response (normalized to their peak ionomycin [FLS] or KCl [neuron] response, [ΔF/Fmax]; cells not crossing threshold for positive controls were excluded from the analysis).

### 2.14. Extraction of RNA-Seq data from rheumatoid arthritis and osteoarthritis patient synovial tissue samples

Publicly available single-cell RNA-Seq data of synovial tissue from 51 OA and RA patients^68^ were used to investigate the presence of proinflammatory genes. For each sample, we extracted the transcripts per million values for the same genes investigated for RT-qPCR of human FLS and organised data by patient ID, cell type, and patient group. The 17 samples that failed or were pending quality control were excluded. The remaining 151 samples were subsequently plotted by dividing into 2 groups—OA and RA.

### 2.15. Statistics

Comparisons between 2 groups with distributed variables were performed using two-sided Student *t*-tests (paired if comparing 2 conditions of the same sample, unpaired otherwise) with suitable corrections and among 3 groups using one-way analysis of variance (ANOVA) followed by Tukey post hoc tests. Proportions were compared for categorical data using χ^2^ tests. Data are shown as mean ± SEM.

## 3. Results

(1) TNF-FLS, but not FLS derived from CFA-induced inflamed knees of mice, have a proinflammatory phenotype and secrete cytokines that activate DRG neurons.

**Figure 1.**
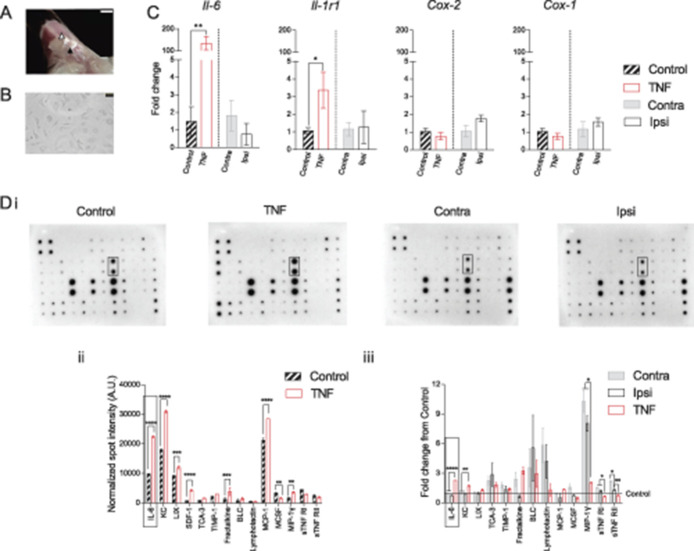
Induction of inflammation in FLS derived from mouse knee. (A) Representative image showing exposed inside of the patella (black triangle) and the surrounding ligament and fat pad (white triangle) after midline resection and distal pull of the quadriceps muscles. Scale bar = 2 mm. (B) Representative image of FLS in culture. Scale bar = 50 µm. (C) Bars represent fold change of the genes *Il-6*, *Il-1r1*, *Cox-2*, and *Cox-1* from either Contra (vs Ipsi) or control (vs TNF). Black hatched bars = control FLS, red bars = TNF-FLS, gray bar = Contra, white bar = Ipsi. All FLS at P5. (D) Images of mouse inflammatory array membranes probed against FLS conditioned medium (i), which were quantified by densitometry and represented as bar graphs showing fold change of various cytokines between control and TNF-FLS (ii, multiple *t* test with Holm–Sidak correction) and among Contra, Ipsi, and TNF from control FLS media (iii, ANOVA with Tukey post hoc test) and spot intensity differences. Rectangles highlight IL-6 spots (Di) and corresponding quantifications (Dii, iii). Only cytokines that were present in all the compared groups are shown in graphs. *P < 0.05, **P < 0.01, ***P < 0.001 and ****P < 0.0001. Error bars = SEM. ANOVA, analysis of variance; FLS, fibroblast-like synoviocytes.

Key genes have been described that act as identifiers of cultured FLS upon isolation by enzymatic digestion of mouse joints,^29^ as well as for establishing their inflammatory phenotype. In this study, we performed RT-qPCR on adherent cells (passages P2-P5) originating from mouse patellae that proliferated in culture (Figs. 1A and B). From P2 to P3, the expression of the macrophage marker^5,29^ cluster of differentiation 68 (*Cd*68, relative to the macrophage cell line RAW 267.4) was significantly reduced, whereas the expression of FLS markers *Cdh11* and *Cd248*^5,29^ remained consistent from P2 to P5 (Supplementary Fig. 1A, B showing expression of FLS marker genes, available at http://links.lww.com/PAIN/A1000); the endothelial marker *Cd31* was not detected (data not shown). These results suggest that from P3, cells cultured from mouse patellae were predominantly FLS and hence subsequent studies were conducted on FLS from P3 to P6.

Concurrently, to establish the proinflammatory phenotype of FLS, we used RT-qPCR to determine the expression of the inflammatory genes *Il-6*, *Il-1r1*, and *Cox-2*, as well as the constitutively expressed gene *Cox-1.* When control FLS (P5, n = 3 biological replicates) were stimulated with 10 ng/mL TNF-α for 48 hours, there was an upregulation of *Il-6* (fold changes: Control, 1.5 ± 0.8 vs TNF-FLS, 135.1 ± 31.1, *P* = 0.006, unpaired *t* test) and *Il-1r1* (fold changes: Control, 1.1 ± 0.2 vs TNF-FLS, 3.4 ± 1.0, *P* = 0.04, unpaired *t* test) expression levels, but not that of *Cox-2* (fold changes: Control, 1.0 ± 0.2 vs TNF-FLS, 0.8 ± 0.2, *P* = 0.2, unpaired *t* test) or *Cox-1* (fold changes: Control, 1.0 ± 0.2 vs TNF-FLS, 0.8 ± 0.2, *P* = 0.18, unpaired *t* test) (Fig. 1C). However, when FLS derived from the inflamed knee (Ipsi, knee width, pre-CFA, 3.1 ± 0.09 mm vs post-CFA, 4.1 ± 0.08 mm, n = 6, *P* = 0.0001, paired *t* test, Supplementary Fig. 1C showing knee width of mice before and after CFA injection, available at http://links.lww.com/PAIN/A1000) of CFA-injected mice were compared to those of the matched contralateral knee (Contra), we did not find any changes in expression levels of the genes between Contra and Ipsi FLS across P2-P5 (Fig. 1C; Supplementary Fig. 1C, available at http://links.lww.com/PAIN/A1000).

We next tested the media (n = 3, each) isolated from control, Contra, Ipsi, and TNF-FLS against a mouse inflammation antibody array (Fig. 1D) to determine the levels of different secreted proinflammatory mediators. This demonstrated that when compared to control FLS media, TNF-FLS media showed presence of regulated on activation, normal T-cell expressed and secreted (RANTES), and granulocyte-macrophage colony stimulating factor (GM-CSF). In addition, TNF-FLS media contained higher levels of IL-6 (*P* < 0.0001), keratinocyte chemoattractant (KC, mouse homolog of IL-8, *P* < 0.0001), lipopolysaccharide-induced chemokine (LIX, *P* < 0.0001), stromal cell-derived factor 1 (SDF-1, *P* < 0.0001), fractalkine (*P* < 0.0001), monocyte chemoattractant protein 1 (MCP-1, *P* < 0.0001), and macrophage inhibitory protein 1γ (MIP-1γ, *P* = 0.002) and lower levels of macrophage colony-stimulating factor (MCSF, *P* = 0.003) compared to control FLS media (multiple *t*-tests with Holm–Sidak corrections, Fig. 1Dii). The TNF-α concentration in TNF-FLS media 48 hours after 10 ng/mL TNF-α stimulation was similar to the control media as determined by the inflammatory antibody array. Mirroring the absence of proinflammatory gene upregulation in Ipsi vs Contra FLS, the spot intensity values of inflammatory cytokines between Ipsi and Contra FLS were similar (multiple *t*-tests with Holm–Sidak corrections, graph not shown), suggesting similar levels of secreted cytokines. Finally, when spot intensities were normalized to control FLS, TNF-FLS media showed increased levels of secreted IL-6 (*P* < 0.0001, ANOVA followed by Tukey multiple comparison test) and KC (*P* = 0.001, ANOVA followed by Tukey multiple comparison test) compared to Ipsi and Contra FLS (Fig. 1Diii).

MIP-1γ was elevated in both Ipsi and Contra FLS when compared to control and TNF-FLS (Fig. 1Diii), perhaps indicating its role in acute, systemic inflammatory pathways. MIP-1γ has been described as having a role in hyperalgesia induced by diabetic neuropathy^49^ and OA-related pain.^19^ We find that MIP-1γ directly activates mouse DRG neurons by producing a dose-dependent Ca^2+^ influx (Supplementary Fig. 1D showing intracellular Ca^2+^ mobilization in DRG neurons upon MIP-1γ application, available at http://links.lww.com/PAIN/A1000), which thus might be a potential mechanism for the in vivo effects described above. However, because MIP-1γ does not have a human homolog (NCBI gene database entry #20308), and thus has limited clinical potential, we did not explore this further.

Taken together, our data suggest that TNF-α stimulation of FLS upregulates expression of canonical inflammatory markers, whereas the knee inflammation observed 24 hours after intra-articular CFA injection does not correlate with a sustained proinflammatory phenotype of FLS isolated from patellae of CFA mice. We also identify other soluble mediators secreted by TNF-FLS that might be involved in FLS-DRG neuron communication. Therefore, given the proinflammatory phenotype of TNF-FLS (and the lack of such phenotype in the FLS expanded from CFA-injected knees), we focused solely on the TNF-FLS for the rest of the study.

(2) TNF-α-stimulated human FLS from OA and RA patients show increased expression of multiple proinflammatory genes that were upregulated in mouse TNF-FLS.

**Figure 2.**
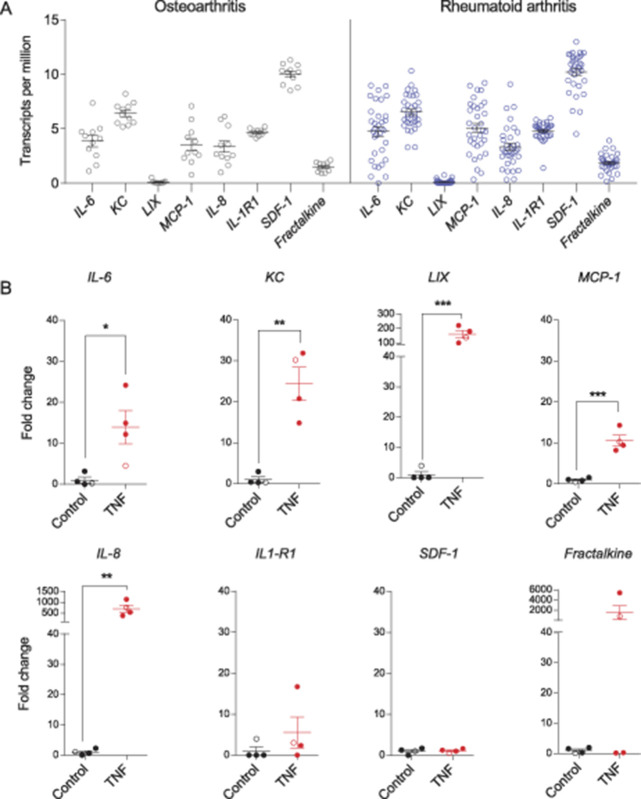
Proinflammatory gene expression in FLS derived from arthritic human patients. (A) Transcripts per million values of selected inflammatory markers extracted from RNA-Seq data of human FLS from RA and OA donors.^68^ Results reveal detectable levels of the proinflammatory genes *IL-6*, *IL-8*, *IL-1R1*, *KC*, *MCP-1*, *SDF-1*, and *Fractalkine* in FLS from both OA (black) and RA (blue) patients. *LIX* was considered undetectable because the mean transcript per million value was below the threshold of 1. (B) Bar graphs showing fold change in expression levels (determined by qPCR) of *IL-6, KC, LIX, MCP-1, IL-8, IL-1R1, SDF-1*, and *Fractalkine* in control vs TNF-α stimulated FLS derived from human RA (filled circles) and OA (open circles) patients (n = 4). *P <0.05, **P <0.01, unpaired *t* test. FLS, fibroblast-like synoviocytes; qPCR, quantitative PCR; RA, rheumatoid arthritis.

To understand whether the identified proinflammatory mediators from mouse FLS are important in human arthritis, we examined gene expression in human FLS. In arthritis, activated FLS contribute to pathogenesis by damaging synovial membranes and secreting inflammatory cytokines, which in turn recruit immune cells.^32,44,45^ In line with these reports, data replotted from a recently published single-cell RNA-seq data set of FLS from 51 OA and RA patients^68^ confirm detectable levels of proinflammatory genes *IL-6, KC, MCP-1, IL-8, IL1-R1, SDF-1,* and *Fractalkine* (Fig. 2A), which matches our mouse data in Figure 1.

Furthermore, to establish whether a similar proinflammatory phenotype can be observed in TNF-α-treated human FLS as in mouse FLS, we used RT-qPCR. When 4 batches of human FLS derived from 4 separate patients were stimulated with 10 ng/mL TNF-α for 24 hours, there was increased expression of *IL-6* (*P* = 0.02), *KC* (*P* = 0.001), *LIX* (*P* = 0.009), *MCP-1* (*P* = 0.0004), and *IL-8* (*P* = 0.005) compared to unstimulated controls (unpaired *t* test) (Fig. 2B). However, expression of *SDF-1* (*P* = 0.8), *IL-1R1* (*P* = 0.3), and *Fractalkine* (*P* = 0.3) was not significantly different in the TNF group compared to control (unpaired *t* test), although for the latter 2 genes, FLS from some patients showed considerable increase after TNF-α stimulation (Fig. 2B).

The data presented here demonstrate the translational potential of our data derived from mouse FLS (Fig. 1), as well as supporting a previous study that showed increased cytokine production from TNF-α-stimulated human FLS from both normal and RA donors compared to unstimulated ones.^32^

(3) TNF-FLS derived from mice increase knee-innervating DRG neuron excitability in coculture.

**Figure 3.**
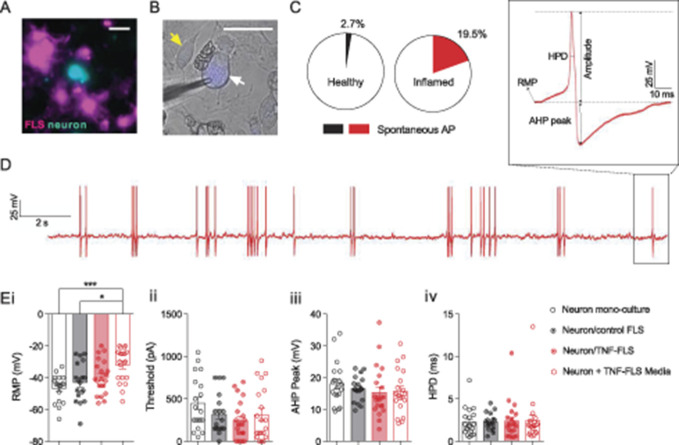
Murine TNF-FLS mediated increase in excitability of knee neurons. (A) Representative live-cell imaging picture showing FLS (magenta) and neuron (cyan) in coculture. Scale bar = 50 µm. (B) Representative FB-labelled knee neuron (white arrow) being recorded using a patch pipette (triangular shadow), surrounded by FLS (yellow arrow). Scale bar = 50 µm. (C) Pie chart showing proportion of knee neurons that fired AP without current stimulation in healthy (neuron monoculture + neuron/control FLS, n = 37, black) and inflamed (neuron/TNF-FLS + neuron/TNF-FLS media, n = 41, red) condition. (D) Representative knee neuron incubated with TNF-FLS media firing spontaneous AP along with schematic diagram of AP properties measured (inset). (E) Bar graphs showing measured RMP (i), threshold (ii), AHP peak (iii), and HPD (iv) from knee neurons in monoculture (n = 19, white bar/black open circle), in coculture with control FLS (n = 18, gray bar/black dotted circle), in coculture with TNF-FLS (n = 20, light red bar/red dotted circle), and incubated in TNF-FLS media (n = 21, white bar/red open circle). *P < 0.05 and ***P < 0.001, ANOVA followed by Tukey post hoc test. Data from 4 to 5 female mice in each group. Error bars = SEM. ANOVA, analysis of variance; FLS, fibroblast-like synoviocytes; RMP, resting membrane potential.

Changes in primary sensory neuron excitability underlie peripheral sensitization, which drives arthritis-related pain.^58^ Nonneuronal cells such as FLS, which are in close proximity with distal terminals of knee neurons, can play instrumental roles in modulating sensory neuron excitability by direct cell contact and/or secretion of proinflammatory cytokines. To determine neuron/FLS communication in health and inflammation, we compared the DRG neuronal excitability of 4 groups: (1) knee neuron monoculture, (2) knee neurons cocultured with control FLS (Figs. 3A and B), (3) knee neurons with FLS that have been exposed to TNF media for 24 hours followed by DRG culture media for 24 hours, and (4) knee neurons with conditioned media from FLS that have been exposed to TNF media for 48 hours to understand the role of soluble mediators in peripheral sensitization. In current-clamp mode, we observed that many neurons spontaneously fired AP during a 20-second recording period (mostly with an intermittent firing pattern, although neurons that fired >100 APs had a continuous firing pattern) in groups 3 and 4. To statistically compare the proportions, we converted our 4-group data into binary categories: neuron monoculture and neuron/control FLS coculture were assigned to the class “healthy,” and neuron/TNF-FLS and neuron/TNF-FLS media were assigned to the class “inflamed.” 19.5% of inflamed neurons (neuron/TNF-FLS, 5/21; neuron/TNF-FLS media, 3/19) fired spontaneous AP compared to 2.7% (neuron monoculture, 0/19; neuron/control FLS, 1/18) of healthy neurons (*P* = 0.02, χ^2^ test). The mean number of APs fired (within the 20 seconds recorded without current injection) by neurons in the neuron/TNF-FLS and neuron/TNF-FLS media categories was 31.6 ± 18.6 and 76.0 ± 62.5, respectively, whereas the neuron in the neuron/control FLS group fired 18 times. This result suggests that there is a general increase in excitability of knee neurons when exposed to an FLS-mediated inflammatory environment (Figs. 3C and D).

On measuring the AP properties (Fig. 3D inset), we found that the resting membrane potential (RMP) was more depolarized in the neuron/TNF-FLS media group compared to both neuronal monoculture (*P* = 0.0004) and neuron/control FLS coculture (*P* = 0.012), which highlights that secreted proinflammatory factors from TNF-FLS likely act upon knee neurons to increase their excitability (ANOVA followed by Tukey post hoc comparison, Fig. 3Ei). This depolarizing trend in RMP was also observed when the data were divided into small and medium-large neurons (Supplementary Fig. 2, available at http://links.lww.com/PAIN/A1000). The other measured properties were unchanged among the 4 groups (ANOVA followed by Tukey post hoc comparison, Table 1 and Figs. 3Eii–iv).

(4) TNF-FLS derived from mice increase TRPV1 function and decrease TRPA1/TRPM8 function of DRG neurons in coculture.

**Table 1:**
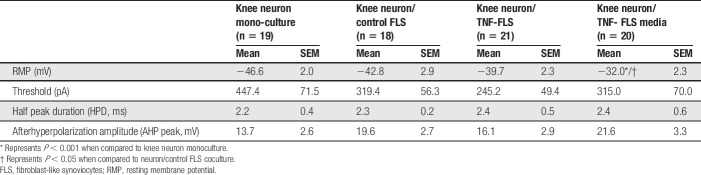
Action potential properties of knee neurons.

**Figure 4.**
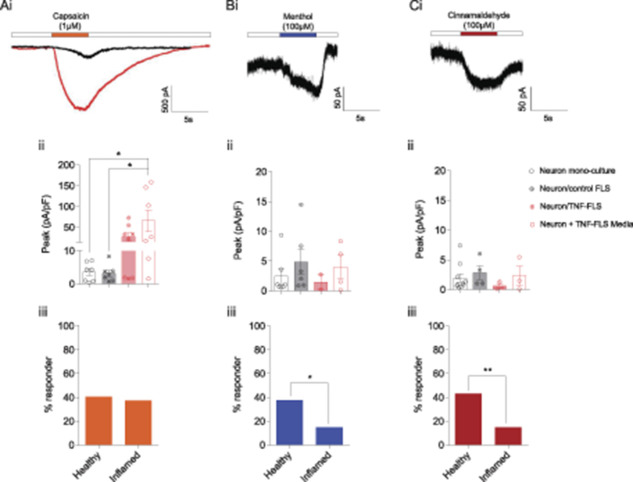
Murine TNF-FLS mediated modulation of TRP agonist response in knee neurons. Representative traces showing capsaicin- (TRPV1 agonist, Ai), cinnamaldehyde- (TRPA1 agonist, Bi), and menthol- (TRPM8 agonist, Ci) evoked responses in knee neurons. Black traces obtained from knee neuron in monoculture, and red trace obtained from knee neuron incubated in TNF-FLS media. White boxes represent perfusion of extracellular solution. Bar graphs showing peak current densities of capsaicin- (Aii), cinnamaldehyde- (Bii), or menthol-evoked (Cii) currents from knee neurons in monoculture (white bar/black open circle), in coculture with control FLS (gray bar/black dotted circle), in coculture with TNF-FLS (light red bar/red dotted circle), and incubated in TNF-FLS media (white bar/red open circle). Comparison between groups made using ANOVA with Tukey post hoc test. Bar graphs showing percent of knee neurons that responded to capsaicin (Aiii), cinnamaldehyde (Biii), and menthol (Ciii) in healthy (neuron monoculture + neuron/control FLS) and inflamed (neuron/TNF-FLS + neuron/TNF-FLS media) condition. Comparison made using χ^2^ test. *P < 0.05. Data from 4 to 5 female mice in each group. Error bars = SEM. ANOVA, analysis of variance; FLS, fibroblast-like synoviocytes.

Dorsal root ganglion neurons in coculture with inflamed FLS derived from AIA rats reportedly have increased expression of TRPV1.^4^ Here, we sought to investigate whether FLS can modulate knee neuron responses to TRP channel agonists using whole-cell patch-clamp recordings. Knee neurons in monoculture and when cocultured with control FLS displayed very similar responses, i.e., a mean capsaicin peak current density response of 3.5 ± 1.0 pA/pF (n = 7/19) and 3.2 ± 0.9 pA/pF (n = 7/18), respectively. By contrast, knee neurons in neuron/TNF-FLS coculture showed a trend for larger magnitude of capsaicin esponses (27.7 ± 9.3 pA/pF, n = 8/21), and those cultured with TNF-FLS media had a capsaicin response of 65.8 ± 25.7 pA/pF (n = 7/20), which was significantly larger in magnitude than neuronal monoculture (*P* = 0.02) and neuron/control FLS coculture (*P* = 0.02) (ANOVA followed by Tukey post hoc comparison, Figs. 4Ai and ii). However, no difference was observed between the percentage of capsaicin responders in healthy (40.5%) vs inflamed groups (37.5%, *P* = 0.8, χ^2^ test, Fig. 4Aiii), suggesting that although TRPV1 channel function is sensitized in TRPV1-expressing neurons, TNF-FLS exposure does not induce TRPV1 expression in neurons lacking any basal expression.

With regards to menthol (monoculture, 2.4 ± 1.2 pA/pF, n = 7/19; neuron/healthy FLS, 4.8 ± 2.1 pA/pF, n = 6/18; neuron/TNF-FLS, 1.4 ± 1.2 pA/pF, n = 2/21; neuron/TNF-FLS conditioned media, 4.0 ± 1.9 pA/pF, n = 4/20; Fig. 4Bi) and cinnamaldehyde (monoculture, 1.9 ± 0.7 pA/pF, n = 10/19; neuron/healthy FLS, 2.8 ± 1.1 pA/pF, n = 4/18; neuron/TNF-FLS, 0.6 ± 0.3 pA/pF, n = 3/21, neuron/TNF-FLS conditioned media, 2.3 ± 1.5 pA/pF, n = 3/20; Fig. 4Ci) responses, all 4 groups showed similar mean peak current density values (Fig. 4Bii and Cii). However, in response to both the TRPA1 (cinnamaldehyde) and TRPM8 (menthol) agonists, the percentage of responding neurons was significantly less in the inflamed group compared to healthy (menthol: healthy vs inflamed, 37.8% vs 15%, *P* = 0.02, χ^2^ test; cinnamaldehyde: healthy vs inflamed, 43.2% vs 15%, *P* = 0.006, χ^2^ test, Figs. 4Biii and Ciii). Taken together, our data suggest that “inflamed” FLS can alter TRP channel agonist response of knee neurons in coculture.

Overall, our data suggest that proinflammatory mediators secreted from FLS are capable of increasing excitability and TRPV1 function of knee-innervating DRG neurons. It has been hypothesized that secretion by FLS is mediated by an increase in intracellular [Ca^2+^]^16,35^ and depolarization. Furthermore, during inflammatory arthritis, FLS are located in an environment with abundant algogens that have been shown to signal through the modulation of TRP channels^9^ and there is also mixed evidence in animals and humans for development of tissue acidosis.^3,24,66^ TRP channels and acid sensors in FLS can increase intracellular [Ca^2+^]^26,35^ and therefore, we tested the sensitivity of mouse knee-derived FLS to a range of acidic pH stimuli, as well as the prototypic TRP channel agonists, capsaicin (TRPV1), cinnamaldehyde (TRPA1), and menthol (TRPM8) using Ca^2+^ imaging. We found that both control and TNF-FLS responded to a range of pH solutions (pH 4.0—7.0) with an increase in intracellular [Ca^2+^], with an increased percentage of TNF-FLS responding to acid than control FLS at pH 5.0 to 6.0 (Supplementary Fig. 3, available at http://links.lww.com/PAIN/A1000). However, capsaicin (control, 3/201, TNF-FLS, 13/252), cinnamaldehyde (control, 1/201, TNF-FLS, 11/252), and menthol (control, 0/201, TNF-FLS, 1/194) evoked a Ca^2+^ response in only a few cells (data not shown), leading us to conclude that very few mouse FLS have functional TRPV1, TRPA1, and TRPM8 ion channels. RT-PCR for control and TNF-FLS revealed expression of multiple proton sensors and low *Trpv1* expression, further validating the Ca^2+^ imaging results (Supplementary Fig. 3, available at http://links.lww.com/PAIN/A1000). Therefore, our data support the hypothesis of Ca^2+^-dependent secretion in FLS leading to increased knee neuron sensitization and nociception.

## 4. Discussion

Fibroblast-like synoviocytes can respond to proinflammatory environments and then themselves become effectors for driving disease pathology, and hence are called “passive responders and imprinted aggressors”.^9^ In support of their activated phenotype, here we demonstrate that FLS obtained from human OA and RA patients and from cell outgrowth of mouse patella can respond to TNF-α stimulation to increase secretion and expression of several proinflammatory mediators. We used these findings to establish a coculture system, which showed that murine FLS and knee neurons can interact, specifically, factors secreted by TNF-FLS increase knee neuron excitability and the magnitude of the response to capsaicin, whereas the proportions of cinnamaldehyde and menthol responding neurons are diminished. To the best of our knowledge, this is the first report to demonstrate FLS-mediated changes in neuronal excitability in coculture and hence directly demonstrate how FLS can regulate articular neurons and in turn arthritis-related pain.

Fibroblast-like synoviocytes from mouse are generally cultured by enzymatically digesting and combining excised joints of forelimbs and hind limbs,^26,29,50,56^ which assumes similarity of FLS derived from all joints. However, a genome-wide study on DNA methylation has shown that important differences exist between knee and hip FLS, including of genes involved in IL-6 signalling.^1^ By using a cell-outgrowth method to culture mouse FLS, as previously described in humans^34^ and rats,^4^ we have avoided the biological ambiguity introduced by joint-to-joint variability. Using these FLS, we investigated the expression of inflammatory genes *Il-6*, *Il-1r1*, and *Cox-2*, all of which have been linked to the inflammatory phenotype of FLS. In brief, stimulating human-derived FLS with IL-1β increases *Cox-2* and *Il-6* expression,^34^ and increased *Il-6* expression is seen in FLS derived from K/BxN mice and after 24-hour stimulation of healthy mouse FLS with TNF-α.^29^ Finally, supernatants from cultured FLS derived from rats 3 days into AIA model show increased IL-6 and PGE_2_.^4^ Here, we observed that neither the expression level of *Il-6*, *Il-1r1,* and *Cox-2*, nor the level of secreted cytokines was upregulated in FLS derived from CFA-injected knee (our local ethics review body did not permit investigation of > 24 hours after CFA injection). This is possibly because the model used here is too brief to influence FLS gene expression, which is supported by a previous study where FLS derived from rats with longer (3-28 days) AIA-induced knee inflammation did have higher PGE_2_ and IL-6 concentrations in culture supernatants compared to control.^4^

Therefore, we created a model of inflammation in vitro where we exposed FLS to TNF-α, a cytokine that is locally upregulated within 3 hours of intraplantar CFA injection in mice.^65^ It is also present in high concentrations in the synovial fluid^36,53^ and tissue of OA and RA patients,^47,61^ along with anti-TNF-α agents being a leading treatment of RA.^60^ We show that TNF-α-stimulated FLS display increased expression of *Il-6* and *Il-1r1* mRNA, with a concomitant increase in secretion of many proinflammatory cytokines, including IL-6—a finding reported previously in human FLS^32^ and replicated in this study. This observation is consistent with FLS being effectors of inflammatory arthritis, and hence we explored the functional repercussions of this proinflammatory phenotype by investigating the ability of “inflamed” FLS to induce functional changes in nerves supplying the knee joint to drive pain.

To understand the effector role of FLS in driving nociception through peripheral sensitization, we set up a coculture system combining FLS and knee neurons. Studying coculture of rat FLS and DRG neurons, von Banchet et al.^4^ showed using immunohistochemistry that bradykinin 2 receptor labelling (but not that of neurokinin 1 or TRPV1) was increased when DRG neurons were cultured with healthy rat FLS, ie, coculture of DRG neurons with FLS can alter expression of genes associated with nociception. Therefore, we first verified that knee neurons in coculture with control FLS do not show dysregulation of excitability or TRP channel agonist response. Then, we asked whether TNF-FLS modulate knee neuron function and found using whole-cell patch clamp that 23% and 16% of knee neurons in neuron/TNF-FLS coculture and neuron/TNF-FLS media (19.5% in the combined “inflamed” condition), respectively, evoked spontaneous AP compared to 6% in neuron/control FLS and 0% in neuron monoculture (2.7% in the combined “healthy” condition). This suggests that TNF-FLS increase the excitability of knee neurons and thus contribute to arthritic pain.

von Banchet et al.^4^ also showed that compared to monoculture, FLS derived from acute and chronic AIA rats induced an increase in TRPV1 protein expression in DRG neurons, which can also lead to sensitization and hence arthritic pain. However, we did not observe an increase in the proportion of neurons responding to capsaicin between healthy and inflamed conditions, which might reflect a species difference and/or difference in knee-specific neuronal population (unlike in this study, von Banchet et al. did not discriminate between knee-innervating neurons and non–knee-innervating neurons); however, we did observe that capsaicin responsive neurons produced larger magnitude responses when incubated with TNF-FLS medium (see below). We also observed that cinnamaldehyde- and menthol-evoked responses were decreased in the inflamed condition, suggesting functional downregulation of TRPA1 and TRPM8, which might compensate for TRPV1 sensitization with regard to overall neuron excitability. Functional downregulation of TRPA1 might be explained through desensitization via increased TRPV1 function^2^ or via an increase in intracellular [Ca^2+^] (due to increased excitability in the inflamed condition).^63^ The latter reason can also be applied to explain decrease of menthol-evoked responses.^52^

We also observed a tendency of a more depolarized RMP and an enhanced magnitude of capsaicin-evoked peak current density of knee neurons in “inflamed” conditions, albeit both only reached statistical significance in the TNF-FLS media incubated group when compared to monoculture and neuron/control FLS. However, we did not observe a decrease in the threshold of AP firing in the inflamed group, despite an increased proportion of these neurons firing spontaneously, thus suggesting perhaps differential susceptibility of certain knee neuron subgroups to TNF-FLS mediated sensitization. For example, a secreted factor may cause one neuronal subpopulation to fire spontaneously (ie, an AP threshold of 0 pA), but overall the average AP threshold would not change due to other neuronal subgroups with a higher threshold not being susceptible to modulation by that secreted factor. Taken together, our data suggest soluble mediators released by FLS are key players in modulating knee neuron excitability, although cell contact with FLS might also play a role in sensitization. We were unable to compare the relative contributions of cell contact and soluble mediators to sensitization because, in this setup, TNF-FLS are always accompanied by TNF-FLS media. We posit that because our experimental design involved a media change 24 hours after TNF incubation of FLS (so as not to directly stimulate neurons with residual TNF-α^18^) in the neuron/TNF-FLS coculture condition, the accumulated soluble mediator concentrations were lower compared to when knee neurons were incubated in 48 hour TNF-FLS conditioned media (no remaining TNF-α was measured in the media at this point). This decrease in concentration of TNF-α is hypothesized to be because of FLS's inability to endogenously secrete TNF-α and/or due to protein degradation.^38^ Our data are consistent with a previous study in FLS cells that measured TNF-α as <4 ng/mL 48 hours after stimulation with 10 ng/ml.^38^ Similarly, in another non–TNF-α-secreting cell line (Clara cells, from airways) 24 hours after initial stimulation with 20 ng/mL TNF-α, the remaining concentration of TNF-α fell below the detection level of the antibody array used.^22^

Indeed, we have recently established the role of soluble mediators present in OA synovial fluid in increasing neuronal excitability and TRPV1 function.^12^ These soluble mediators mainly consist of cytokines/chemokines, which form complex signalling pathways with neurons (reviewed in Refs 43,^54^). In this study, we have identified several of them to be upregulated in TNF-FLS media, which have been reported to be able to directly signal to neurons: IL-6,^23^ KC,^10^ RANTES,^46^ GM-CSF,^21^ LIX,^42^ SDF-1,^46^ MCP-1,^33^ and MIP-1γ (this study). In the case of IL-6, MCP-1, and GM-CSF, there have also been reports of an increase in TRPV1 function.^21,23,33^

In summary, we have established a coculture system to provide evidence for a direct inflammation-pain axis through FLS and DRG neurons. In the future, it can be adapted to investigate FLS-mediated peripheral sensitization using FLS obtained from chronic models of arthritis, activated by other proinflammatory cytokines or arthritic synovial fluid.

## Conflict of interest statement

The authors have no conflicts of interest to declare.

## Appendix A. Supplemental digital content

Supplemental digital content associated with this article can be found online at http://links.lww.com/PAIN/A1000.
